# Progress of Section “Bioorganic Chemistry and Medicinal Chemistry”

**DOI:** 10.3390/cimb48010035

**Published:** 2025-12-26

**Authors:** Hidayat Hussain

**Affiliations:** International Joint Laboratory of Medicinal Food Development and Health Products Creation, Biological Engineering Technology Innovation Center of Shandong Province, Heze Branch of Qilu University of Technology (Shandong Academy of Sciences), Heze 274000, China; hussainchem3@gmail.com

*Current Issues in Molecular Biology* (*CIMB*) (https://www.mdpi.com/journal/cimb, accessed on 24 December 2025) was established in 1999. The journal’s “Bioorganic Chemistry and Medicinal Chemistry” (BCMC) section was established in 2021, and has published 300 articles to date, including 104 in 2025 alone. The guiding objective of the “Bioorganic Chemistry and Medicinal Chemistry” section is to expeditiously disseminate contributions seeking to elucidate the molecular mechanisms behind the deleterious effects of compounds in cells, animals, populations, and the environment, or in pertinent models. Another purpose of this section is to publish research addressing biological questions and queries at the molecular level, with the employment of organic chemistry, medicinal chemistry, and molecular chemistry. Topics covered by this section include natural products and synthetic molecules with biological effects at the molecular level, medicinal chemistry, computational chemistry, enzyme catalysis and transformation, enzyme inhibition, biomimetic chemistry, and biological probes.

The section has achieved undoubtedly well-deserved maturity and visibility, as evidenced by the large number of high-quality publications across all fields associated with bioorganic chemistry and related areas (medicinal chemistry, chemical biology, molecular biology, etc.). Research groups from around the world have made valuable contributions to a broad range of topics over the past year by publishing original articles and reviews of considerable interest. Given the significant number of papers that have been published, it is possible to highlight only a few of the most relevant developments covering the section’s main research areas.

In 2022, cancer, a major worldwide health problem, caused around 9.7 million deaths worldwide. Among women, breast cancer remains the most common form of malignant tumour diagnosed [1]. Despite the progress made in diagnostics and therapeutics, preventive strategies have become increasingly important because they are more accessible and can reduce cancer risk [2]. The olive tree (*Olea europaea* L.), particularly its leaves, has been used in traditional medicine around the world for many years, and has been shown to have a range of biological effects, particularly anticancer effects [3,4,5,6,7,8,9,10,11,12,13]. Systemic antioxidant capacity and immune regulation are enhanced by olive leaf tea (OLT) supplementation, which in turn contributes to tumour prevention and organ protection. The authors’ findings suggest that OLT is of natural origin, safe and bioactive, making it a promising nutraceutical candidate for cancer therapy, either as a preventative measure or as an additional treatment [14].

Another interesting review outlines the positive biological impacts of curcumin, particularly its anticancer effects, and details its mechanisms and progress through preclinical and clinical trials [15]. A particular focus is placed on recent progress in nanoformulation strategies that improve how curcumin is absorbed by the body and how it is delivered to specific areas. In addition, the growing trend of combination therapy is investigated, where curcumin works with chemotherapeutics and phytochemicals in a way that is stronger than the sum of the combination’s parts when it comes to overcoming drug resistance and enhancing the effectiveness of anticancer drugs.

Breast milk’s effectiveness can be attributed to the presence of various bioactive molecules that have been shown to protect against infections [16], enhance immune function [17], reduce inflammation [18], and influence the infant microbiome [19]. In an intriguing article, Trofin et al. [20] draw parallels between the immune response triggered by natural SARS-CoV-2 infection and that induced by the COVD-19 vaccine, offering valuable insights into the nature and quality of the immunity transferred to infants through breast milk. The examination of a series of biologically active substances within the breast milk of infected or vaccinated mothers demonstrated an augmentation of passive immunity, thereby fortifying the infants’ immune system and plausibly diminishing the likelihood of infections, including those caused by the novel coronavirus.

Diabetes mellitus is regarded as one of the most severe healthcare issues of recent times, and rising numbers of diabetic patients are a clear indication of its prevalence. The data shows a projected increase from 537 million in 2021 to 783 million by 2045 [21]. Alfadda et al. [21] studied differences in the urinary proteome in patients with type 2 diabetes and the patients’ protein profile using mass spectrometry. Their investigations revealed that, following a twelve-week treatment period with liraglutide, the patients exhibited enhanced glycemic control and a modified urinary protein profile. The authors hypothesised that the augmentation of MT-2 and diminution of α1-AT and ZAG could be attributable to the renoprotective effect of liraglutide. In another interesting article, Gadewar et al. [22] evaluated the antidiabetic effects of Solanum indicum fruit extract in diabetic rats. The experimental results confirmed that the fruit extract had two effects: it lowered blood glucose levels and substantially lowered elevated blood cholesterol and triglyceride levels. In addition, the prevention of oxidative stress associated with type II diabetes in STZ rats was achieved.

Finally, I would like to draw your attention to a thorough review that outlines the positive impacts of coffee and its polyphenols on human health [23]. This subject has been a source of much debate and disagreement, and around which consensus has yet to emerge, which indicates how necessary it is to examine this topic in greater depth. Numerous bioactive compounds have been reported in coffee and have shown a range of potential beneficial effects [24,25,26,27,28,29,30]. The review of the evidence this article provides highlights the complicated role played by coffee and its bioactive ingredients in the modulation of inflammation, bone metabolism, and oxidative stress—key processes in the pathophysiology of periodontitis. High doses of caffeine have been linked to a decrease in bone formation and calcium levels; however, the polyphenolic compounds found in coffee, particularly chlorogenic acid, have shown strong anti-inflammatory, antioxidant, and osteoprotective properties in both in vitro and in vivo studies [23]. 

Expectations are high for significant progress in the field of molecular informatics from 2026 onwards. With more and more molecular data available, and with improvements in AI tools and machine learning, it is thought that more accurate predictions of molecular behaviour and better drug discovery processes will soon be possible. Another trend is the ‘safe-by-design’ strategy. Heretofore, the potential dangers associated with a novel chemical or product were addressed at comparatively late stages in its research and development process of such, frequently when the product was on the verge of being launched onto the market. Adopting a holistic approach from the outset of a product’s life cycle enables us to proactively ‘design out’ potential hazards. When dealing with innovations that carry significant biochemical, societal, and ecological uncertainties, the ‘safe-by-design’ strategy is particularly important.

The BCMC section of the journal receives a great many submissions and will carry on publishing excellent research. The journal’s impact factor has increased in recent years, as have the number of citations of published papers and the overall number of submissions. These factors indicate that the journal is valued by the scientific community worldwide. However, as editors, we should strive to improve the workflow and the quality of the published papers.

To sum up, the BCMC section at CIMB is dedicated to the ongoing publication of top-tier articles in the field. This commitment aims to make a substantial contribution to the international scientific and medical community, thereby advancing the well-being of humanity. As editors, we will ensure that the journal clearly focuses on medicinal chemistry and drug discovery, and that the published research meets the highest standards of originality, scientific rigour, and relevance to the field. A more rigorous peer-review process is to be implemented by us as editors, with multiple rounds of review by subject matter experts to improve the quality of published papers. Many fields are yet to be explored, including: (a) important enzyme inhibitory studies associated with numerous diseases, (b) detailed mechanistic studies of novel natural products, (c) enzymatic synthesis of bioactive molecules, (d) elucidation of biosynthetic pathways of bioactive substances, and (e) new therapeutics with new mechanisms to treat diabetes, cancer, Alzheimer’s disease, and other dangerous human diseases.
